# Construction of Metal/Zeolite Hybrid Nanoframe Reactors via *in-Situ*-Kinetics Transformations

**DOI:** 10.1021/acscentsci.4c00439

**Published:** 2024-05-24

**Authors:** Ge Tian, Guangrui Chen, Guoju Yang, Zhenheng Diao, Risheng Bai, Ji Han, Buyuan Guan, Jihong Yu

**Affiliations:** † State Key Laboratory of Inorganic Synthesis and Preparative Chemistry, College of Chemistry, Jilin University, Changchun 130012, People’s Republic of China; § International Center of Future Science, Jilin University, Changchun 130012, People’s Republic of China; ∥ School of Chemical Engineering, Changchun University of Technology, Changchun 130012, People’s Republic of China

## Abstract

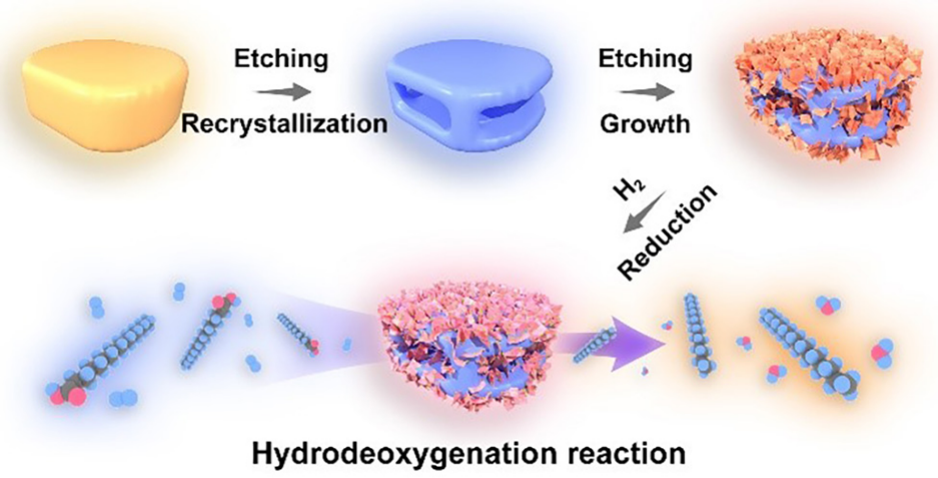

Metal/zeolite hybrid nanoframes featuring highly accessible compartmental environments, abundant heterogeneous interfaces, and diverse chemical compositions are expected to possess significant potential for heterogeneous catalysis, yet their general synthetic methodology has not yet been established. In this study, we developed a two-step *in-situ*-kinetics transformation approach to prepare metal/ZSM-5 hybrid nanoframes with exceptionally open nanostructures, tunable metal compositions, and abundant accessible active sites. Initially, the process involved the formation of single-crystalline ZSM-5 nanoframes through an anisotropic etching and recrystallization kinetic transformation process. Subsequently, through an *in situ* reaction of the Ni^2+^ ions and the silica species etched from ZSM-5 nanoframes, layered nickel silicate emerged on both the inner and outer surfaces of the zeolite nanoframes. Upon reduction under a hydrogen atmosphere, well-dispersed Ni nanoparticles were produced and immobilized onto the ZSM-5 nanoframes. Strikingly, this strategy can be extended to immobilize a variety of ultrasmall monometallic and bimetallic alloy nanoparticles on zeolite nanoframes. Benefiting from the structural and compositional advantages, the resultant hybrid nanoframes with a high loading of discrete Ni nanoparticles exhibited enhanced performance in the hydrodeoxygenation of stearic acid into liquid fuels. Overall, the methodology shares fresh insights into the rational construction of intricate frame-like metal/zeolite hybrid nanoreactors for many potential catalytic applications.

## Abstract 2

Metal/zeolite nanoframes with highly open nanostructure and discrete metal nanoparticles are constructed via a general *in-situ*-kinetic transformation method for the hydrodeoxygenation of stearic acid.

## 1 Introduction

Multifunctional catalysts play a pivotal role in various chemical processes due to their ability to perform multiple functions simultaneously or in a sequential manner.^1−5^ Bifunctional catalysts, combining metal and acid sites, are commonly employed to facilitate sequential reactions due to their ability to catalyze a broad but specific set of reactions.^6−8^ As a typical class of metal–acid bifunctional catalysts, zeolite-supported metal catalysts find extensive use in various industrial reactions,^3,9,10^ such as hydrocracking,^11^ hydroalkylation,^12,13^ isomerization,^14−16^ and hydrogenation/dehydrogenation.^17,18^ In general, the metal nanoparticles could be supported on zeolites by impregnation or ion exchange methods. However, the metal ions cannot fully penetrate micropores and aggregate easily on the outer surface of the zeolite, leading to large and nonuniform metal nanoparticles. Recently, the ligand-protected method has been reported to construct zeolite-supported metal catalysts, showing outstanding thermal stability and catalytic performance.^21,22^ However, the limited loading amount of metals and the inevitable use of organic additives might hinder its large-scale industrial application. Furthermore, the narrow micropores of the resultant zeolite-supported metal catalysts typically limit the mass transfer process, and bulky substrate molecules are unable to reach the metal sites within the zeolite framework through the microporous channels, leading to an unsatisfactory catalytic efficiency. Considering these notable limitations encountered by zeolite-supported metal catalysts, hierarchically porous zeolite-supported metal catalysts with improved mass-transfer efficiency, elevated surface area,^23−26^ and heightened accessibility to both metal and acid active sites have gained preference for practical applications.^27−31^

Nanoframe-structured catalysts have been demonstrated to be ideal candidates for catalytic applications due to their notable surface area-to-volume ratios, efficient molecular accessibility, and nanoconfinement effect.^32−35^ Recently, by exploiting the cooperative effect of anisotropic etching and recrystallization, ZSM-5 nanoarchitectures with controllable open configurations and chemical compositions have been successfully prepared.^36^ Metal/zeolite hybrid nanoframes with a hierarchical porosity and high metal dispersion are anticipated to demonstrate remarkable catalytic performance by integrating functional metal nanoparticles with significantly open zeolite nanoarchitecture. However, the synthetic methodology of metal/zeolite hybrid nanoframes has not yet been established. Therefore, establishing a general synthetic method to achieve an even dispersion of various metal or alloy nanoparticles on zeolite nanoframes holds significant promise in producing high-performance zeolite-supported metal catalysts.

**Figure 1:**
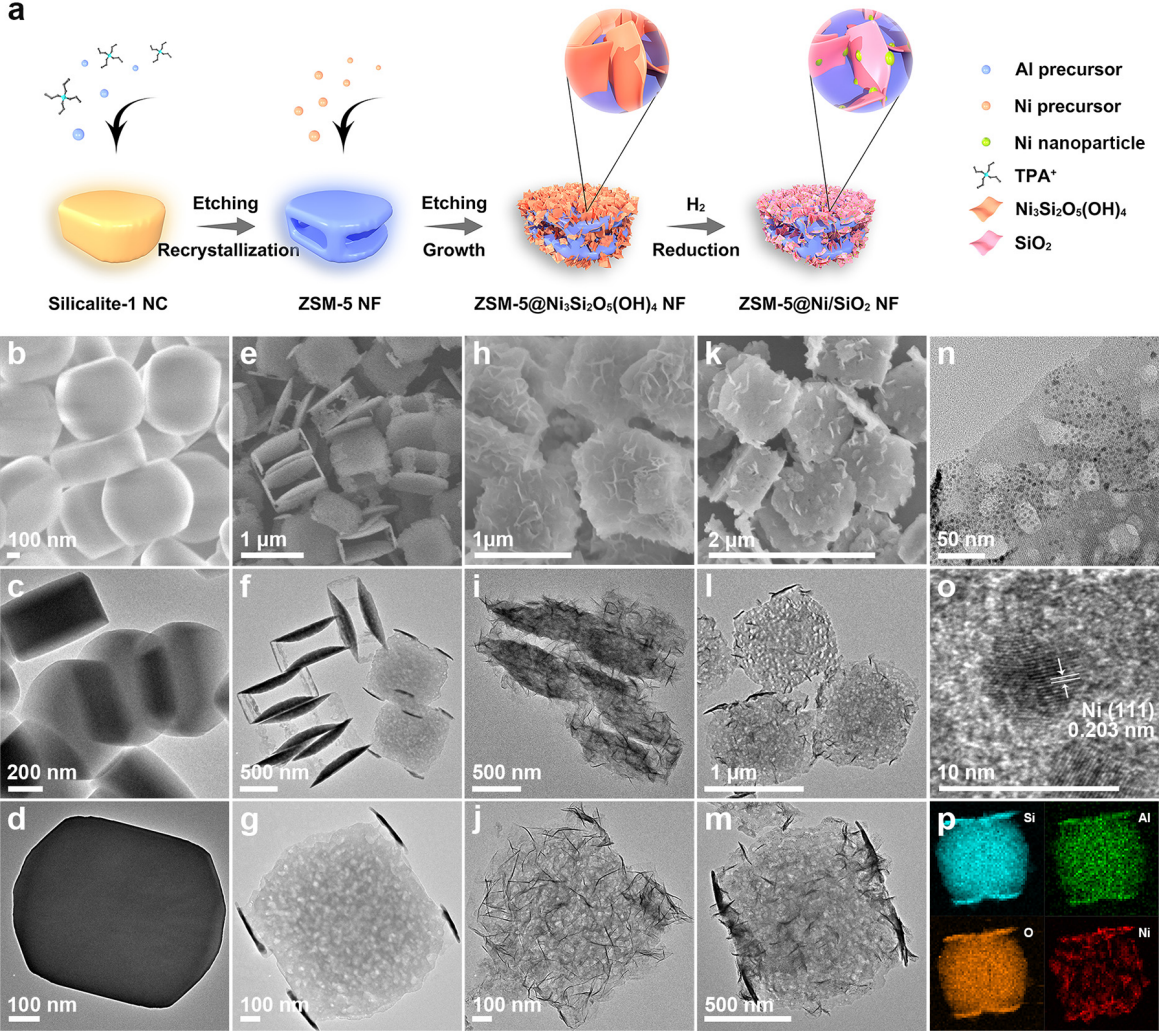
(a) Schematic illustration of the synthetic process. (b, e, h, k) SEM and (c, d, f, g, i, j, l–n) TEM images of (b–d) silicalite-1 NCs, (e–g) ZSM-5 NFs, (h–j) ZSM-5@Ni_3_Si_2_O_5_(OH)_4_ NFs, and (k–n) ZSM-5@Ni/SiO_2_ NFs. (o) High-resolution TEM image of a Ni nanoparticle and (p) elemental mapping images of a ZSM-5@Ni/SiO_2_ NF.

Here, we report a general *in-situ*-kinetics transformation strategy for the introduction and stabilization of high-loading Ni nanoparticles on hierarchically porous ZSM-5 nanoframes (NFs, Figure 1a). Silicalite-1 nanocrystals (NCs) are employed as the templates for the synthesis of ZSM-5 NFs. By employing a two-stage etching process involving isotropic and anisotropic etching of silicalite-1 templates, alongside simultaneous *in situ* recrystallization of ZSM-5 on dynamically evolving zeolite templates in alkaline media, the kinetic process triggers an anisotropic transformation from solid zeolite particles into frame-like nanoarchitectures with hierarchical porosity (Figure S1). Furthermore, the second transformation process results in the creation of ZSM-5 NFs enveloped with layered Ni_3_Si_2_O_5_(OH)_4_ nanosheets (denoted as ZSM-5@Ni_3_Si_2_O_5_(OH)_4_ NFs) via a subsequent reaction involving the Ni^2+^ ions and silica species *in situ* etched from the ZSM-5 nanoframes. After reduction in a hydrogen atmosphere, the layered Ni_3_Si_2_O_5_(OH)_4_ on the ZSM-5 nanoframes is transformed into highly dispersed Ni nanoparticles embedded in the amorphous SiO_2_ matrix (denoted as ZSM-5@Ni/SiO_2_ NFs). The approach facilitates the controlled preparation of ZSM-5@Ni/SiO_2_ NFs with tailored Ni loading. Moreover, it can also be expanded to produce various monometallic or bimetallic alloy nanoparticle-loaded ZSM-5 nanoframes. Owing to the highly open frame-like nanostructures and high-loading Ni nanoparticles, the obtained ZSM-5@Ni/SiO_2_ nanoframe catalysts exhibit enhanced performance in the hydrodeoxygenation (HDO) of stearic acid to liquid fuels.

## 2 Results and Discussion

Silicalite-1 NCs are prepared through a conventional hydrothermal reaction. Scanning electron microscopy (SEM) and transmission electron microscopy (TEM) images demonstrate the uniformity of the silicalite-1 templates with an average size of approximately 600 nm (Figure 1b–d). After the initial *in-situ*-kinetics transformation in the presence of aluminum isopropoxide and tetrapropylammonium hydroxide (TPAOH) for 24 h at 170 °C, silicalite-1 NCs are transformed into ZSM-5 NFs. Figure 1e shows a typical SEM image of the as-formed ZSM-5 NFs with a three-dimensional open frame-like nanostructure. TEM image confirms the existence of a cavity and through macroporous holes within the shell along the [001] and [100] directions (Figure 1f). Close observation of an individual ZSM-5 NF at a higher magnification unveils the presence of irregular mesopores within the zeolite frame’s nanoshell (Figure 1g). The ZSM-5@Ni_3_Si_2_O_5_(OH)_4_ NFs are synthesized by a subsequent *in-situ*-kinetics transformation of ZSM-5 nanoframes in the presence of Ni^2+^ ions under alkaline media. Figure 1h depicts that the sample retains its frame-like architecture, but the surface exhibits a significantly rougher texture. The nanostructure of the ZSM-5@Ni_3_Si_2_O_5_(OH)_4_ NFs is further elucidated by TEM. Each hybrid nanoframe shows a frame-like structure adorned with nanosheet subunits present on both their inner and outer surfaces (Figure 1i). A closer examination of the nanosheet subunits reveals that the thickness of the Ni_3_Si_2_O_5_(OH)_4_ sheets is only ∼4 nm (Figure 1j). The structural evolution from ZSM-5 NFs to ZSM-5@Ni_3_Si_2_O_5_(OH)_4_ NFs is tracked by SEM (Figure S2). After annealing the sample in H_2_ at an elevated temperature, ZSM-5@Ni/SiO_2_ NFs can be obtained. As revealed from SEM and TEM images (Figure 1k–m), the structure of ZSM-5@Ni/SiO_2_ nanoframes remains nearly unchanged compared to their precursors. The magnified TEM image shows the Ni nanoparticles are highly dispersed with an average size of ∼4.5 nm (Figure 1n). The (111) lattice fringes of metallic Ni with an interplanar distance of 0.203 nm can be clearly observed (Figure 1o). As revealed by elemental mapping results, Si, Al, Ni, and O elements are evenly distributed throughout a ZSM-5@Ni/SiO_2_ hybrid nanoframe (Figure 1p).

**Figure 2:**
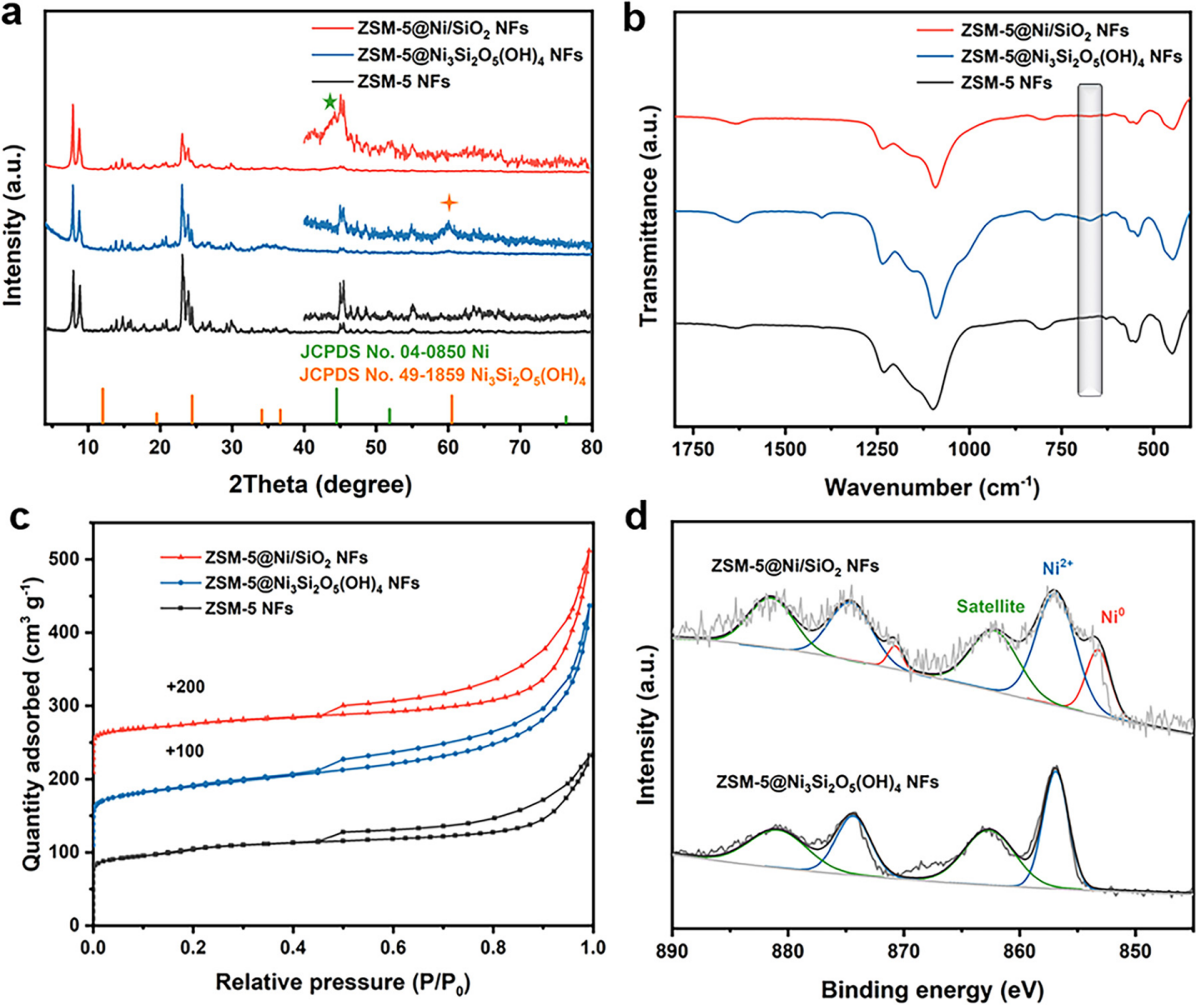
(a) XRD patterns, (b) FTIR spectra, and (c) N_2_ adsorption/desorption isotherms of ZSM-5 NFs, ZSM-5@Ni_3_Si_2_O_5_(OH)_4_ NFs, and ZSM-5@Ni/SiO_2_ NFs. (d) Ni 2p XPS spectra of ZSM-5@Ni_3_Si_2_O_5_(OH)_4_ NFs and ZSM-5@Ni/SiO_2_ NFs.

Powder X-ray diffraction (XRD) analysis discloses that both silicalite-1 NCs and ZSM-5 NFs exhibit distinct characteristic peaks of a typical **MFI** structure (Figure 2a and Figure S3). Following a hydrothermal treatment in an aqueous solution of nickel acetate, the resulting ZSM-5@Ni_3_Si_2_O_5_(OH)_4_ NFs exhibit an additional diffraction peak at approximately 60.5° in the high-angle region, which can be indexed as the (060) planes of Ni_3_Si_2_O_5_(OH)_4_. Following H_2_ reduction, ZSM-5@Ni/SiO_2_ shows the characteristic peak of metallic nickel at 44.5°. The result indicates that the Ni species in layered nickel silicate are converted into metallic Ni nanoparticles. Fourier transform infrared (FTIR) spectroscopy is used to identify structural and bonding information on as-synthesized nanoframes. Compared with ZSM-5 NFs, a new band emerges at around 670 cm^–1^ for ZSM-5@Ni_3_Si_2_O_5_(OH)_4_ NFs, which is ascribed to the Si–O–Ni stretching vibrations. This band vanishes upon the formation of ZSM-5@Ni/SiO_2_ NFs after H_2_ reduction, because the layered nickel silicate nanosheets undergo transformation into dispersed Ni nanoparticles and amorphous SiO_2_ nanosheets (Figure 2b). The ^27^Al magic-angle spinning nuclear magnetic resonance (MAS NMR) spectroscopy exhibits the chemical shift of 58 ppm in ZSM-5 NFs and ZSM-5@Ni_3_Si_2_O_5_(OH)_4_ NFs, which is attributed to the tetrahedrally coordinated aluminum in their frameworks (Figure S4). Temperature-programmed reduction of the hydrogen (H_2_-TPR) profile of the ZSM-5@Ni_3_Si_2_O_5_(OH)_4_ NFs is shown in Figure S5. The major reduction peak ranging from 400 to 705 °C is assigned to the nickel silicate species. The other minor peak, observed in the range of 320 to 360 °C, is likely attributable to the nickel hydroxide species.^37^ N_2_ sorption measurements reveal a variation in pore information among the samples (Figure 2c). Different from the microporous matrix of silicalite-1 NCs, ZSM-5 NFs, ZSM-5@Ni_3_Si_2_O_5_(OH)_4_ NFs, and ZSM-5@Ni/SiO_2_ NFs show hierarchically porous features (Figure S6). X-ray photoelectron spectroscopy (XPS) reveals the valence states of Ni species (Figure 2d). The as-synthesized ZSM-5@Ni_3_Si_2_O_5_(OH)_4_ NFs exhibit prominent signals of Ni^2+^ at 856.8 eV. After calcination under the H_2_ atmosphere, a combination of metallic Ni^0^ and Ni^2+^ is discernible on the surface of ZSM-5@Ni/SiO_2_ NFs. This phenomenon can be attributed to the easy oxidation of the metallic Ni nanoparticles to form NiO layers on their surfaces when exposed to air.^38^

**Figure 3:**
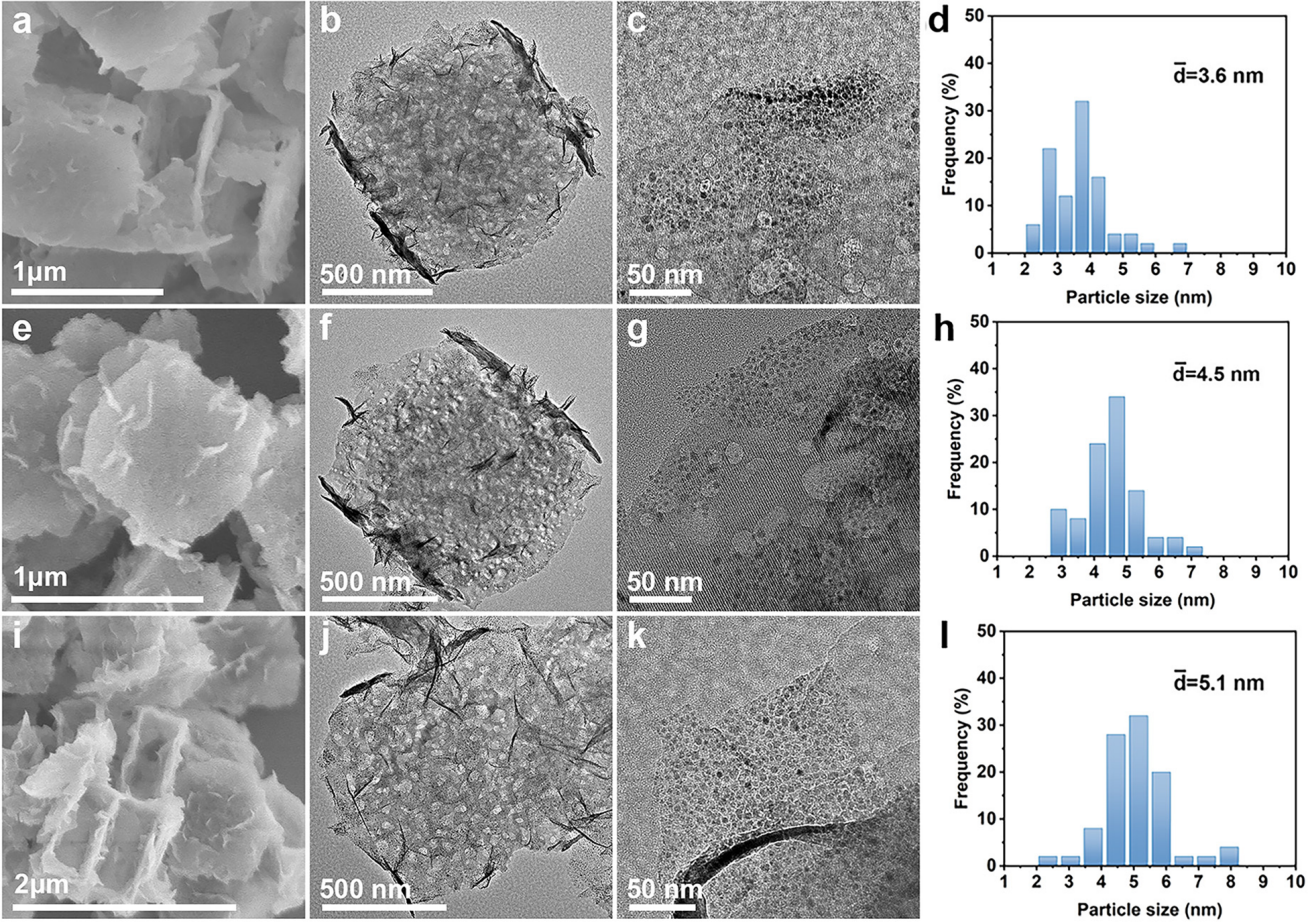
(a, e, i) SEM and (b, c, f, g, j, k) TEM images of ZSM-5@Ni/SiO_2_ NFs with Ni loading of (a–c) 7.8 wt %, (e–g) 14.5 wt %, and (i–k) 17.1 wt % as well as (d, h, l) the corresponding size distributions of Ni nanoparticles.

The Ni loading of ZSM-5@Ni/SiO_2_ NFs can be precisely tailored by tuning the amount of ZSM-5 NFs added to the reaction systems. By decreasing the amount of ZSM-5 NFs from 20 mg to 10 mg, then to 8 mg, the number of nickel silicate nanosheets on the surface of ZSM-5 NFs increases (Figure 3a,b,e,f,i,j). After reduction under the H_2_ atmosphere, the Ni nanoparticles are well dispersed and uniformly distributed across the silica nanosheets on the ZSM-5 NFs (Figure 3c,g,k). The metal loadings are 7.8%, 14.5%, and 17.1%, respectively, as determined by inductively coupled plasma optical emission spectroscopy (ICP-OES) analysis. The average sizes of Ni nanoparticles are increased from 3.6 nm to 4.5 nm, then to 5.1 nm (Figure 3d,h,l). XRD patterns also confirm the characteristic peaks of Ni_3_Si_2_O_5_(OH)_4_ and Ni nanoparticles (Figures S7 and S8).

**Figure 4:**
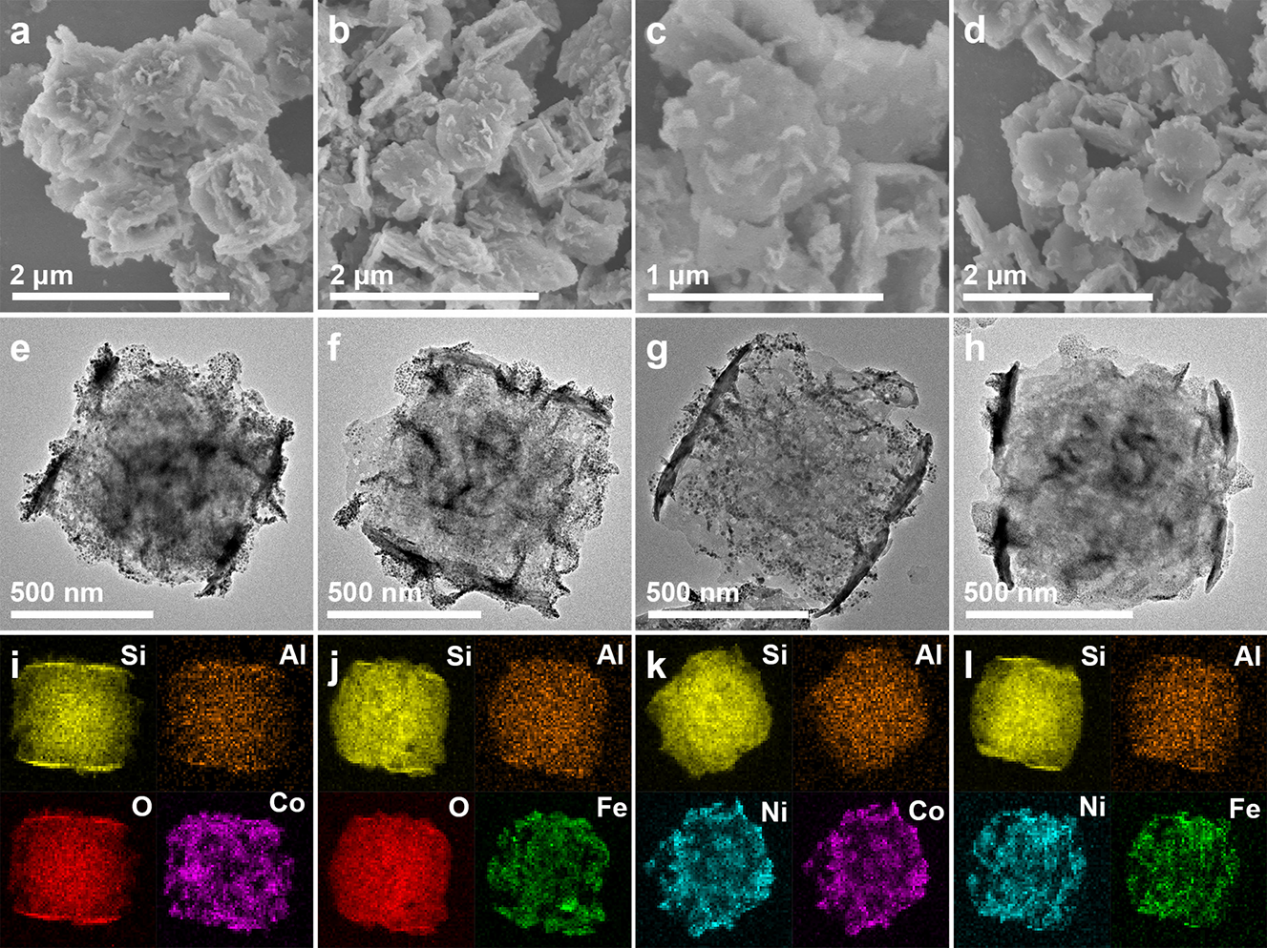
(a–d) SEM, (e–h) TEM, and (i–l) elemental mapping images of (a, e, i) ZSM-5@Co/SiO_2_ NFs, (b, f, j) ZSM-5@Fe/SiO_2_ NFs, (c, g, k) ZSM-5@Ni-Co/SiO_2_ NFs, and (d, h, l) ZSM-5@Ni-Fe/SiO_2_ NFs.

Furthermore, we have demonstrated the versatility of this approach by successfully synthesizing various ZSM-5 nanoframe-supported monometallic and bimetallic alloy nanoparticles. Various ZSM-5@metal silicate NFs (metal = Co, Fe, Ni–Co, and Ni–Fe) can be successfully synthesized by similar synthetic procedures except that different metal precursors are used instead (Figures S9–S12 and Table S1). After reduction under the H_2_ atmosphere, ZSM-5@metal/SiO_2_ NFs (metal = Co, Fe, Ni–Co alloy, Ni–Fe alloy) are formed (Figure 4a–h). In all cases, the frame-like structure of the zeolite supports can be well maintained after the reduction. Elemental mappings (Figure 4i–l) indicate the dispersion of metal nanoparticles on ZSM-5 NFs. The XRD patterns indicate that all the hybrid nanoarchitectures display distinctive peaks associated with the corresponding metals and the **MFI** framework (Figures S13–S16).

**Figure 5:**
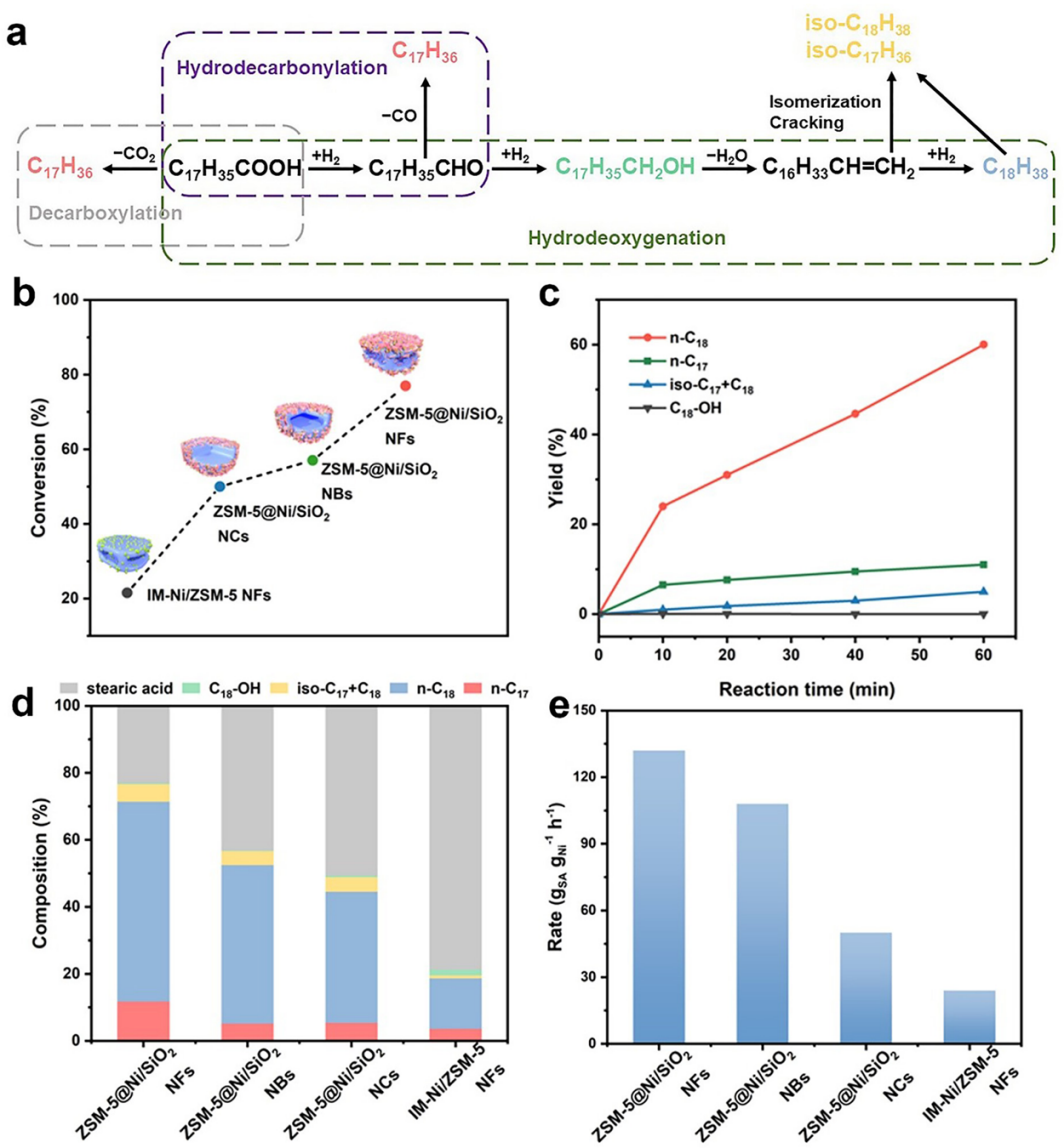
(a) A schematic illustration depicting the hydrodeoxygenation reaction of stearic acid for biomass upgrading processes. (b) Conversion of stearic acid over various catalysts. (c) The yield for stearic acid HDO over ZSM-5@Ni/SiO_2_ NFs. (d) Product compositions for stearic acid HDO over various catalysts. (e) Comparison of normalized rates for the hydrodeoxygenation of stearic acid over the various catalysts.

The hydrodeoxygenation of stearic acid was employed as the model reaction for the conversion of bioderived lipids into renewable transportation fuels (Figure 5a). The hydrogenation of the carboxyl group in stearic acid at metal sites leads to the formation of 1-octadecanol, which can be transformed into octadecene through acid-catalyzed dehydration. Subsequently, hydrogenation of octadecene occurs at the metal site, resulting in the formation of octadecane. Additionally, heptadecane can be obtained through the metal-catalyzed decarboxylation of stearic acid or the hydrodecarbonylation of octadecanal. The acidic sites in the zeolite induce hydroisomerization and hydrocracking of alkanes, resulting in the production of iso-octadecane and iso-heptadecane.^39−41^ In general, the narrow micropores in zeolites impose diffusion limitations on large stearic acid molecules, thus restricting accessibility to internal acid and embedded metal sites within conventional metal/zeolite catalysts. Therefore, the ZSM-5@Ni/SiO_2_ NFs are expected to show improved catalytic performance because of excellent mass transfer efficiency and accessible abundant bifunctional metal/acid sites.^42,43^ Besides ZSM-5@Ni/SiO_2_ NFs, the Ni-loaded ZSM-5 nanocrystals (ZSM-5@Ni/SiO_2_ NCs), Ni-loaded ZSM-5 nanoboxes (ZSM-5@Ni/SiO_2_ NBs, Figure S17), and impregnation-prepared Ni-loaded ZSM-5 nanoframes (IM-Ni/ZSM-5 NFs) with similar Ni content and Si/Al ratio were used as reference catalysts for comparative analysis. All reference catalysts were examined by XRD measurement, and only peaks corresponding to the **MFI** framework and Ni nanoparticles were observed (Figure S18). N_2_ sorption analysis of the reference catalysts reveals capillary condensation steps at low relative pressure and hysteresis loops within the relative pressure range of 0.45–0.99, indicating the coexistence of micropores and mesopores (Figure S19).^44^ Furthermore, all the reference catalysts possess similar metal loadings of ∼14 wt %, as measured by ICP-OES analysis.

The electron microscopy images of ZSM-5@Ni/SiO_2_ NCs, ZSM-5@Ni/SiO_2_ NBs, and IM-Ni/ZSM-5 NFs show that the average sizes of Ni nanoparticles are 6.0, 4.5, and 20.1 nm, respectively (Figures S20–S22). The metal dispersion measured from CO pulse chemisorption is shown in Table S2. The Ni dispersion of ZSM-5@Ni/SiO_2_ NFs synthesized through thermochemical reduction of metal silicate is 16%, significantly higher than that of IM-Ni/ZSM-5 NFs (1%) prepared by the impregnation–reduction approach. The layered metal silicate facilitates the immobilization and homogeneous dispersion of metal species, thus resulting in the formation of dispersed metal nanoparticles on silica nanosheets after the thermal reduction process. FTIR studies of pyridine (Py) and 2,6-di-*tert*-butylpyridine (DTBP) sorption were employed to quantify the acidity of samples. Specifically, 35% of the acid sites in ZSM-5@Ni/SiO_2_ NFs are identified as external acid sites, whereas for ZSM-5@Ni/SiO_2_ NCs, the percentage of external acid sites is 12% (Table S3).

All the catalysts are evaluated in the conversion of biomass to fuel by hydrodeoxygenation of stearic acid at 260 °C with 4 MPa of H_2_. The stirring speed is set to 1000 rpm to eliminate external mass transport limitations (Figure S23). The conversion of stearic acid can reach 77% in 1 h for the ZSM-5@Ni/SiO_2_ NFs (Figure 5b and Figure S24). In contrast, the ZSM-5@Ni/SiO_2_ NBs and ZSM-5@Ni/SiO_2_ NCs show lower conversion rates of 57% and 50%, respectively, in the same period. The improved catalytic activity can be attributed to the presence of a highly open nanostructure that can expose more active acid sites in zeolite for the dehydration of alcohols. Moreover, the conversion over the ZSM-5@Ni/SiO_2_ NFs synthesized by two-step *in-situ*-kinetics transformation processes is much higher than that over the IM-Ni/ZSM-5 NFs prepared by the impregnation process. The variation in catalytic activity between ZSM-5@Ni/SiO_2_ NFs and IM-Ni/ZSM-5 NFs may be attributed to the higher dispersion of Ni nanoparticles in ZSM-5@Ni/SiO_2_ NFs compared to those in IM-Ni/ZSM-5 NFs, thereby exposing more active sites for the conversion of reactant. The analysis of liquid product distribution using ZSM-5@Ni/SiO_2_ NFs (Figure 5c) shows n-octadecane (n-C_18_) as the predominant product, accounting for 59.6% of the yield. There is also a relatively lower selectivity observed for n-heptadecane (n-C_17_) with the yield of 11.8%. These results suggest that the primary pathway for hydrodeoxygenation of stearic acid involves the hydrogenation of fatty acids into their corresponding fatty alcohols, followed by subsequent alcohol dehydration and further hydrogenation. In addition, 5.3% alkane isomers (iso C_17_+C_18_) are produced with ZSM-5@Ni/SiO_2_ NFs as catalyzed by the acid sites. As further revealed by the kinetics of the intermediate product 1-octadecanol, the conversion rate of 1-octadecanol exceeds that of stearic acid for all the catalysts, indicating that Ni-catalyzed stearic acid hydrogenation is the rate-determining step in the overall reaction (Figure S25 and Table S4). The ZSM-5@Ni/SiO_2_ sample also exhibits the highest yield of alkanes up to 76.7% among all catalysts (Figure 5d), compared to those of the ZSM-5@Ni/SiO_2_ NBs (56.7%), ZSM-5@Ni/SiO_2_ NCs (48.9%), and IM-Ni/ZSM-5 NFs (19.6%). To gain deeper insights into the relationship between various nanostructures of catalysts and their internal diffusion restrictions, the normalized reaction rates are calculated with eq S1 to compare their catalytic activities (Figure 5e). By altering their nanostructures from solid nanocrystals to nanoboxes and eventually to nanoframes, we observed changes in the thickness of the microporous layers, decreasing from 310 nm to 40 nm and then to 25 nm (Figure S26). As expected, the conversion rate of stearic acid follows the order ZSM-5@Ni/SiO_2_ NCs (50 g_SA_ g_Ni_^–1^ h^–1^) < ZSM-5@Ni/SiO_2_ NBs (108 g_SA_ g_Ni_^–1^ h^–1^) < ZSM-5@Ni/SiO_2_ NFs (132 g_SA_ g_Ni_^–1^ h^–1^). IM-Ni/ZSM-5 NFs consisting of much bigger metal nanoparticles show the lowest stearic acid conversion rate of 24 g_SA_ g_Ni_^–1^ h^–1^. The above result confirms that the open structures of ZSM-5@Ni/SiO_2_ NFs with more accessible acid sites and well-dispersed Ni nanoparticles successfully alleviate diffusion constraints for large stearic acid molecules, leading to enhanced activity in the hydrodeoxygenation reaction.

## 3 Conclusion

In summary, a general *in-situ*-kinetics transformation strategy has been developed to anchor highly dispersed monometallic and bimetallic alloy nanoparticles (metal = Ni, Co, Fe, Ni–Co alloy, and Ni–Fe alloy) on ZSM-5 nanoframes. The key feature of this novel approach involves employing zeolite nanoframes coated by metal silicates with high metal loading contents as the precursors for creating metal/zeolite bifunctional catalysts, which could ensure the immobilization of well-dispersed metal nanoparticles onto the zeolite nanoframes. The metal loading of the metal/zeolite hybrid catalysts can be facilely modulated by adjusting the amount of zeolite nanoframes added in the reaction systems. As compared with conventional zeolite-supported metal catalysts, the metal/zeolite hybrid catalysts show remarkably decreased sizes of metal species, efficient mass transport efficiency, and abundant accessible active sites. As a proof of concept, Ni-loaded ZSM-5 nanoframes are utilized for the hydrodeoxygenation of stearic acid. The high activity and selectivity exhibited by the Ni-loaded ZSM-5 nanoframes in the hydrodeoxygenation of stearic acid highlight the considerable potential of zeolite nanoframe-supported metal catalysts for diverse catalytic applications.

## Supporting Information Available

Experimental procedure details and methods, XRD patterns, H_2_-TPR, SEM images, TEM images, nitrogen sorption isotherms, and catalytic tests of different metal/zeolite hybrid nanostructures (PDF)
