# Supplementary material for: Construction of Metal/Zeolite Hybrid Nanoframe Reactors via in-Situ-Kinetics Transformations
Source: ACS Cent Sci. 2024 May 24;10(8):1473–80. doi: 10.1021/acscentsci.4c00439 (PMC11363334; doi:10.1021/acscentsci.4c00439)
Supplement: Supplementary file 1 — oc4c00439_si_001.pdf [file oc4c00439_si_001.pdf]

Supporting Information

Construction of Metal/Zeolite Hybrid Nanoframe Reactors via *In-Situ*-Kinetics Transformations

Ge Tian,[†,‡] *Guangrui Chen,*[†,§,‡] *Guoju Yang,*[†] *Zhenheng Diao,*[†,||] *Risheng Bai,*[†] *Ji Han,*[†] *Buyuan Guan,*[*,†,§] *and Jihong Yu*[*,†,§]

[†]State Key Laboratory of Inorganic Synthesis and Preparative Chemistry, College of Chemistry, Jilin University, Changchun 130012, P. R. China

[§]International Center of Future Science, Jilin University, Changchun 130012, P. R. China

[||]School of Chemical Engineering, Changchun University of Technology, Changchun 130012, P. R. China

*Correspondence to: guanbuyuan@jlu.edu.cn; jihong@jlu.edu.cn

This PDF file includes 24 pages, Figures S1 to S26, and Tables S1 to S4.

Table of Contents

Section 1. Materials and Methods

Section 2. Characterizations

Section 3. Catalytic tests

Section 4. Additional figures and tables

Section 1. Materials and Methods

Tetraethylorthosilicate (TEOS, Tianjin Fuchen Chemical Reagents Company), tetrapropylammonium hydroxide solution (TPAOH, 25 wt.%, Sinopharm Chemical Reagent Company), ammonia solution (25%-28%, Sinopharm Chemical Reagent Company), aluminium isopropoxide ($Al(C_3H_7O)_3$, Tianjin Fuchen Chemical Reagents Company), nickel acetate ($Ni(CH_3COO)_2 \cdot 4H_2O$, Sinopharm Chemical Reagent Company), ammonium chloride (NH_4Cl, Sinopharm Chemical Reagent Company), cobalt nitrate hexahydrate ($Co(NO_3)_2 \cdot 6H_2O$, Sinopharm Chemical Reagent Company), iron(II) sulfate heptahydrate ($FeSO_4 \cdot 7H_2O$, Sinopharm Chemical Reagent Company), $Ni(NO_3)_2 \cdot 6H_2O$ (Sinopharm Chemical Reagent Company), sodium aluminate ($NaAlO_2$, Sinopharm Chemical Reagent Company), sodium hydroxide (NaOH, Sinopharm Chemical Reagent Company), stearic acid. All reagents were used without further purification. Deionized (DI) water was used in all experiments.

Synthesis of Silicalite-1 nanocrystals (NCs). The molar composition of the mixture was: 1 SiO_2: 0.27 TPAOH: 112 H_2O. Typically, 1.2 ml of TPAOH (25 wt%) was mixed with 10 ml of water, and then 1.2 ml of TEOS was added to the solution. The mixture was stirred continuously for 4 h. The obtained clear solution was transferred into a 20 ml Teflon-lined stainless steel autoclave and crystallized at 170 °C for 12 hours in a rotational oven. The white product was separated by centrifugation, washed with water and ethanol several times, dried at 60 °C in the oven overnight, and then calcinated at 550 °C for 6 h.

Synthesis of ZSM-5 nanoframes (NFs). Briefly, 2 mg of aluminum isopropoxide was added into 10 ml of 0.22 M TPAOH solution, and then 120 mg of silicalite-1 nanocrystals was dispersed into the mixture by ultrasonication. The suspension was then transferred into an autoclave and crystallized at 170 °C for 24 h in a rotational oven. The product was separated by centrifugation, washed with water and ethanol several times, and then dried at 60 °C in the oven overnight, followed by calcination at 550 °C for 6 h.

Synthesis of ZSM-5@$Ni_3Si_2O_5(OH)_4$ NFs. ZSM-5@$Ni_3Si_2O_5(OH)_4$ NFs was prepared through a simple hydrothermal process. Typically, 8.7 mg of $Ni(CH_3COO)_2{\cdot}4H_2O$, 37.4 mg of NH_4Cl, and 63.7mg of $NH_3{\cdot}H_2O$ (28%) were added under stirring in 3.5 g of distilled water. 20 mg of the as-prepared ZSM-5 NFs was then added to the above solution and ultrasonicated for 30 min to form a uniform suspension, then the mixture was heated to 100 °C for 3 h. The resulting green precipitates were collected and washed several times with distilled water and ethanol. The final products were dried at 60 °C in the oven for 4 h.

Synthesis of ZSM-5@Ni/SiO_2 NFs. ZSM-5@$Ni_3Si_2O_5(OH)_4$ NFs were reduced under a H_2 flow (flow rate: 20 ml min^{-1}) at 550 ^{o}C for 0.5 h with a heating rate of 5 ^{o}C min^{-1}, and black power of ZSM-5@Ni/SiO_2 NFs was collected.

Synthesis of ZSM-5 NCs. To synthesize ZSM-5 NCs, 1.22g of TPAOH solution (25 wt.%), 20 mg of sodium hydroxide, and 60 mg of sodium aluminate were dissolved in 4 g of deionized

water. Then, 2.083 g of TEOS was added to the mixture and vigorously stirred for 6 h. Finally, hydrothermal treatment was carried out at 170 °C for 72 h under static condition. The H-type ZSM-5 NCs were prepared via an ion-exchange process. The as-prepared ZSM-5 NCs were dispersed in 100 ml of 1.0 M NH_4Cl solution at 80 °C for 8 h three times. Afterward, the obtained product was washed with deionized water several times and calcined in a muffle furnace at 550 °C for 6 h.

Synthesis of ZSM-5 nanoboxes (NBs). Briefly, 1.219 g of TPAOH solution (25 wt%), 40 mg of sodium hydroxide, and 20 mg of sodium aluminate were dissolved in 3.4 ml of water at room temperature under stirring. Then, 2.083 g of TEOS was added to the mixture and vigorously stirred for 6 h. Finally, hydrothermal treatment was carried out at 170 °C for 72 h under static condition. The H-type ZSM-5 was obtained through the same the ion-exchange and annealing process. To prepare H-type ZSM-5 NBs, 1 g of H-type ZSM-5 was dispersed in 10 ml of 0.2 M TPAOH solution. The mixture was heated in an autoclave at 170 °C under stirring for 3 d. The product was separated by centrifugation, washed with water and ethanol several times, and then dried at 60 °C in the oven overnight, followed by calcination at 550 ºC for 6 h.

Synthesis of ZSM-5@Ni/SiO_2 NCs and ZSM-5@Ni/SiO_2 NBs. ZSM-5@Ni/SiO_2 NCs and ZSM-5@Ni/SiO_2 NBs were prepared under a similar experimental process as ZSM-5@Ni/SiO_2 NFs, with the only variation being the use of ZSM-5 NCs and ZSM-5 NBs instead of ZSM-5

NFs.

Synthesis of IM-Ni/ZSM-5 NFs. In a typical synthesis, 0.1 g of $Ni(NO_3)_2 \cdot 6H_2O$ was dissolved in 1 g of distilled water, and then the solution was slowly dropped onto 1 g of ZSM-5 NFs with continuous stirring at ambient temperature for a total of 4 h. Afterwards, the material was firstly dried overnight at 80 ^oC for 12 h. Then the catalyst precursor was calcined in air (flow rate: 100 ml min^{-1}) at 400 ^oC for 4 h and reduced under a H_2 flow (flow rate: 20 ml min^{-1}) at 500 ^oC for 5 h with a heating rate of 2 ^oC min^{-1} .

Synthesis of ZSM-5@metal silicate NFs (metal = Co, Fe, Ni-Co, Ni-Fe). ZSM-5@metal silicate NFs (Metal=Co, Fe, Ni-Co, Ni-Fe) were prepared by post synthesis and ion exchange. 10 mg of ZSM-5@$Ni_3Si_2O_5(OH)_4$ NFs was treated in 4 ml of aqueous solution containing 4 mmol of NH_3, 2 mmol of NH_4Cl, 0.27 mmol of N_2H_4, 0.10−0.25 mmol of trisodium citrate, and 0.07−0.25 mmol of $FeSO_4 \cdot 7H_2O$ or $Co(NO_3)_2 \cdot 6H_2O$ at 80−90 °C for 2−6 h. The products were separated by centrifugation and washed with water. Detailed synthesis conditions for ZSM-5@metal silicate NFs are summarized in Table S1.

Synthesis of ZSM-5@metal/SiO_2 NFs (metal = Co, Fe, Ni-Co alloy, Ni-Fe alloy). ZSM-5@metal/SiO_2 NFs were obtained by reduction of the corresponding ZSM-5@metal silicate NFs under a H_2 flow at 400 ^oC for 0.5 h.

Section 2. Characterizations

The powder X-ray diffraction measurements were performed on a Rigaku D-Max 2550 diffractometer by using Cu Kα radiation. Scanning electron microscopy images were measured with JEOL JSM-6700F. The transmission electron microscopy images and the elemental mappings were measured with a Tecnai F20 electron microscope. The H_2-TPR measurements were measured on a Micromeritics AutoChem II 2920 instrument. Chemical compositions of samples were analyzed by inductively coupled plasma (ICP) using Perkin-Elmer Optima 3300 DV ICP instrument. Nitrogen adsorption/desorption measurements were carried out on a Micromeritics ASAP 3-flex analyzer at 77 K after the samples were degassed at 350 °C under vacuum. XPS spectra of the catalysts were performed using a Thermo ESCALAB 250 spectrometer (Thermo Scientific, NY, USA). Fourier transform infrared (FTIR) spectra were recorded on a BRUKER vertex 80v; samples before testing were pelleted with KBr powder. Fourier transform infrared (FT-IR) spectra of pyridine (Py) and 2,6-di-*tert*-butyl-pyridine (DTBP) analysis were recorded on Bruker Tensor 27. All samples were prepared as a self-supported wafer and placed inside an IR transmission cell and then pretreated under vacuum at 450 °C for 1 h followed by cooling to 150 °C. Py or DTBP was adsorbed onto the sample for 30 min at 150 °C, and the mixture was evacuated for 1 h before the spectrum was recorded. CO pulse chemisorption was performed by a Micromeritics AutoChem 2920. Before the test, 100 mg of catalyst was activated in a flow of 100 ml min^{-1} 10 vol% H_2 in He at 500 °C for 2 h and then blew with He for 1 h. After cooling to 40 °C, the CO gas pulses (5 vol% in He) were introduced at a flow rate of 100 ml min^{-1}. The changes in the CO gas phase concentration were recorded by TCD.

Section 3. Catalytic tests

The deoxygenation reaction of stearic acid (SA) was carried out in a batch autoclave (CHEMN Instrument, 100 ml). In a typical run, 0.1 g of catalysts, 1 g of SA and 40 ml of dodecane were introduced into the batch autoclave. The autoclave was sealed and firstly purged with N_2 (30 bar) three times to remove the residual air, followed by filling it with the reaction gas H_2 (40 bar) at room temperature. The reaction was performed at 260 °C at a stirring speed of 1000 rpm. The liquid products were obtained by *in-situ* sampling every 20 min and analyzed by gas chromatography (GC, Agilent 7890B) equipped with HP-innowax column (30 m × 320 μm × 25 μm) and FID detector. The methyl heptadecanoate was used as a quantitative internal standard in GC measurement. The mass balance was above 98%.

The yield, conversion, and normalized rate were calculated based on the following equations:

The normalized rate ($g_{SA}\ g_{Ni}^{-1}\ h^{-1}$) = mass of converted SA (g)/mass of nickel in the catalyst (g)/reaction time (h) (Equation S1)

The conversion (%) = mass of the converted SA (g)/mass of the starting SA (g) ×100% (By GC analysis) (Equation S2)

The selectivity (%) = mass of one product (g)/mass of all the products (g) ×100% (By GC analysis) (Equation S3)

The yield (%) = conversion×selectivity×100% (Equation S4)

Section 4. Additional figures and tables

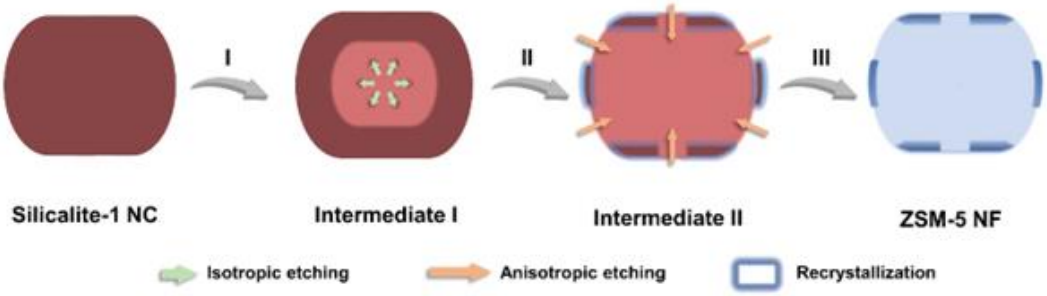

Figure S1. The formation mechanistic details of ZSM-5 nanoframe via *in-situ*-kinetics transformation. Stage I: isotropic etching. Stage II: simultaneous anisotropic etching and recrystallization. Stage III: intracrystalline ripening.

Note: The interior defect-rich part of the silicalite-1 NC is first isotropically etched by exposure to OH^- ions at the beginning of the reaction to produce a nanobox with uniform shell thickness. As the reaction time proceeds, the etching rate becomes faster at the corners along the [101] direction and the central part of {100} facets along the [100] direction. Meanwhile, the organic structure-directing agent (OSDA) and inorganic precursors coassemble on the dynamically evolving zeolite templates. With the intracrystalline ripening process proceeding, the ZSM-5 NF with a three-dimensional open frame-like structure is formed.

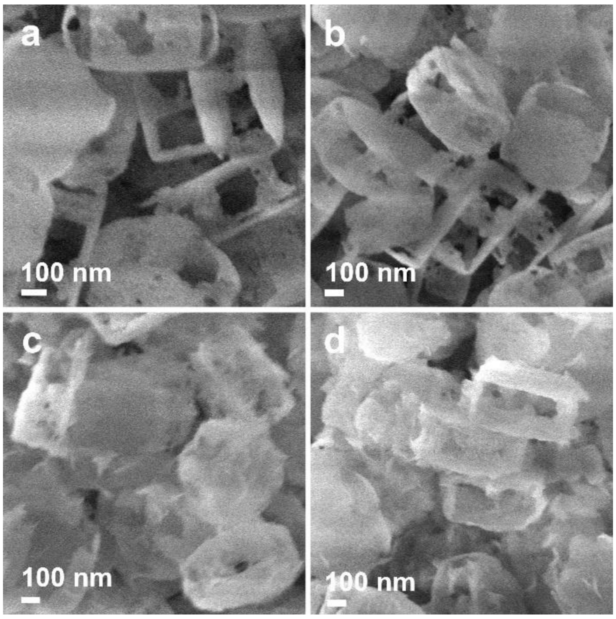

Figure S2. SEM images of ZSM-5@$Ni_3Si_2O_5(OH)_4$ NFs collected at different reaction times: (a) 0.5 h, (b)1 h, (c) 2 h, and (d) 3 h.

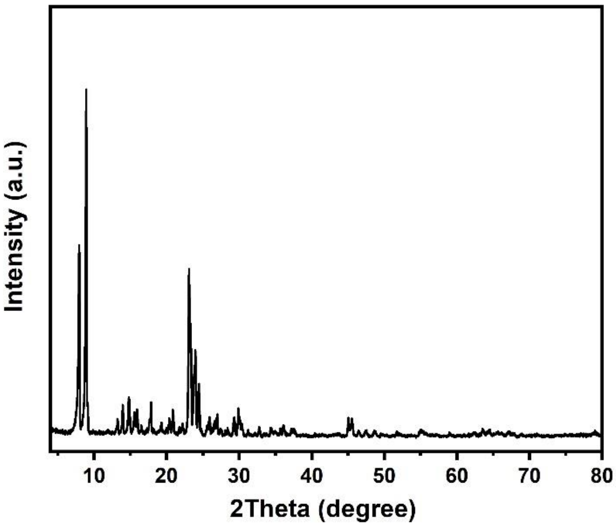

Figure S3. XRD pattern of silicalite-1 NCs.

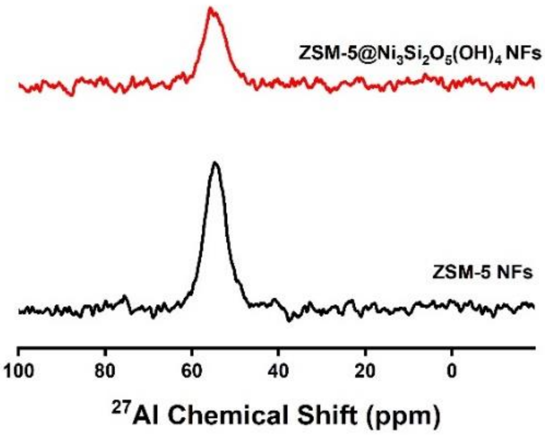

Figure S4. ^{27}Al MAS NMR spectra of ZSM-5 NFs and ZSM-5@$Ni_3Si_2O_5(OH)_4$ NFs.

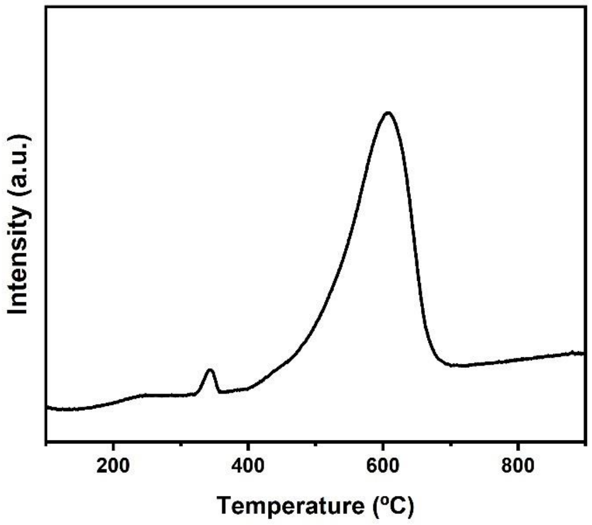

Figure S5. H_2-TPR profile of ZSM-5@$Ni_3Si_2O_5(OH)_4$ NFs.

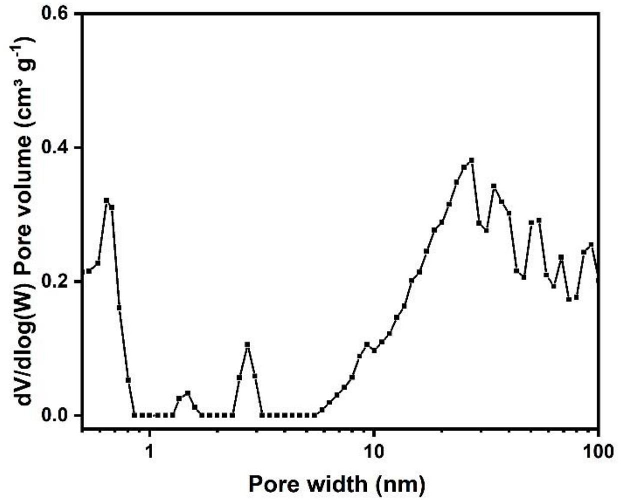

Figure S6. The pore size distribution of ZSM-5@Ni/SiO_2 NFs.

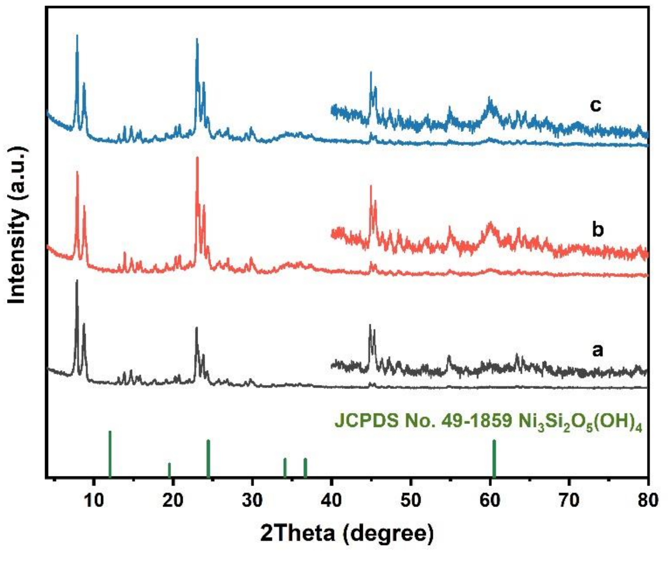

Figure S7. XRD patterns of ZSM-5@$Ni_3Si_2O_5(OH)_4$ NFs with the nickel loading of (a) 7.8 wt%, (b) 14.5 wt%, and (c) 17.1 wt%.

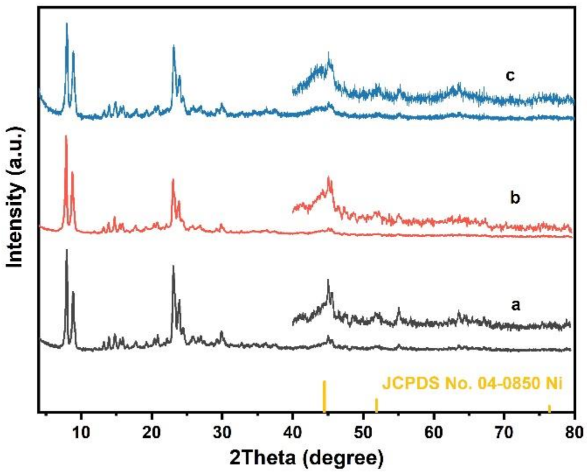

Figure S8. XRD patterns of ZSM-5@Ni/SiO_2 NFs with nickel loading of (a) 7.8 wt%, (b) 14.5 wt%, and (c) 17.1% wt%.

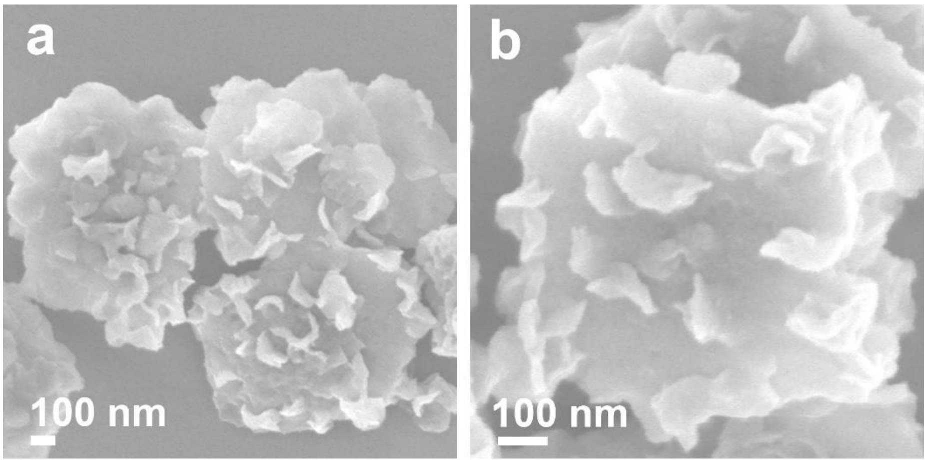

Figure S9. SEM images of ZSM-5@Co silicate NFs.

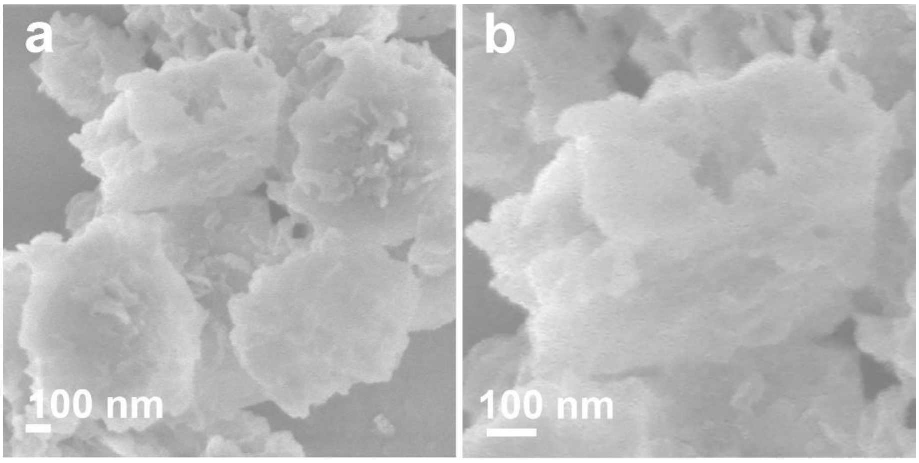

Figure S10. SEM images of ZSM-5@Fe silicate NFs.

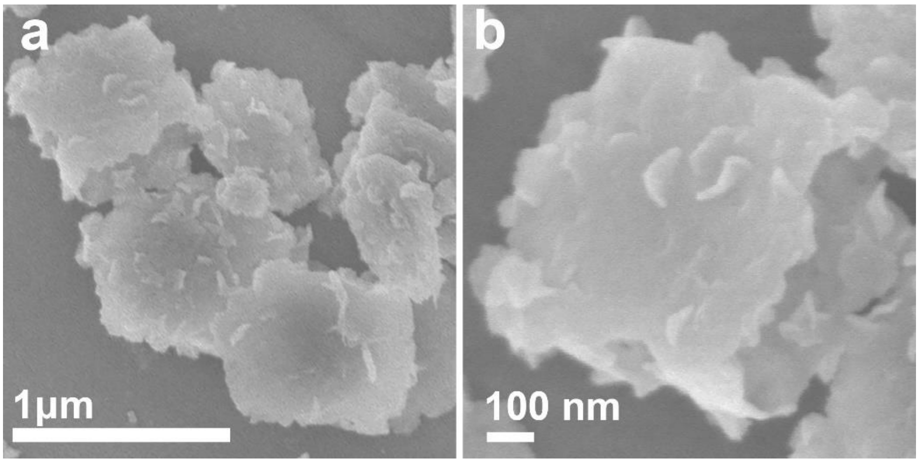

Figure S11. SEM images of ZSM-5@Ni-Co silicate NFs.

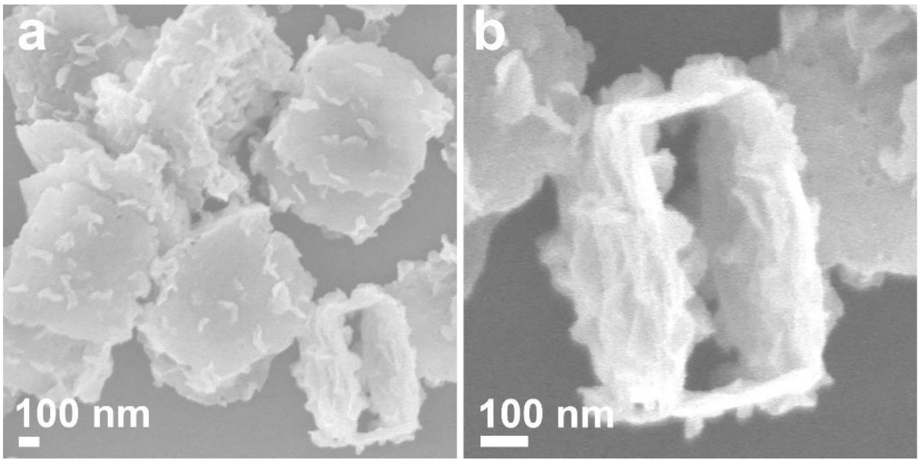

Figure S12. SEM images of ZSM-5@Ni-Fe silicate NFs.

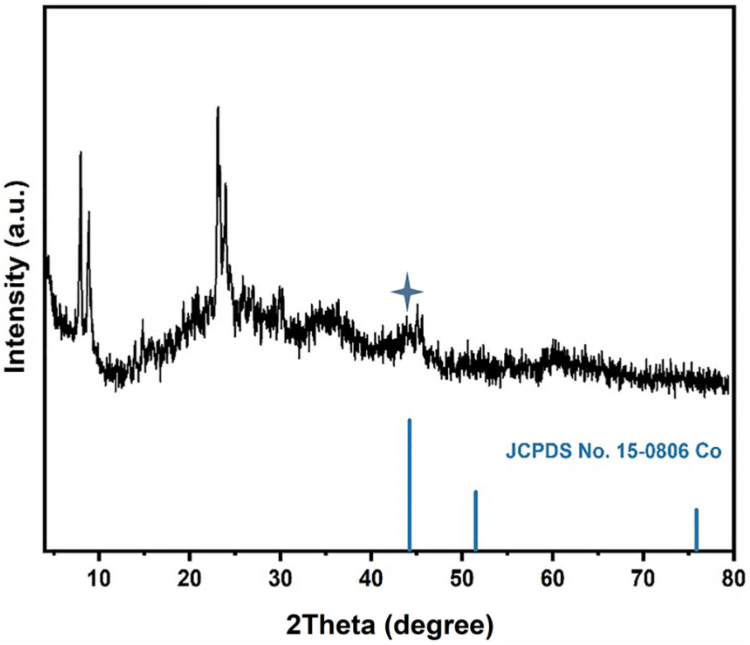

Figure S13. XRD pattern of ZSM-5@Co/SiO_2 NFs.

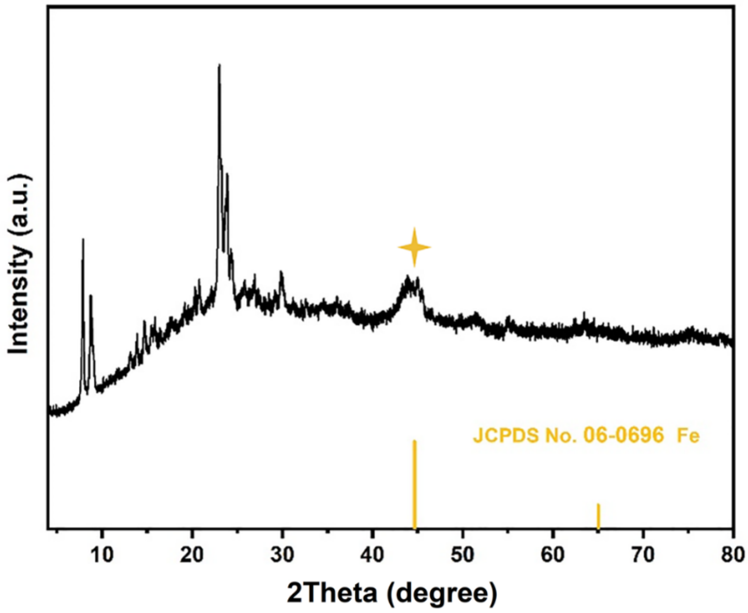

Figure S14. XRD pattern of ZSM-5@Fe/SiO_2 NFs.

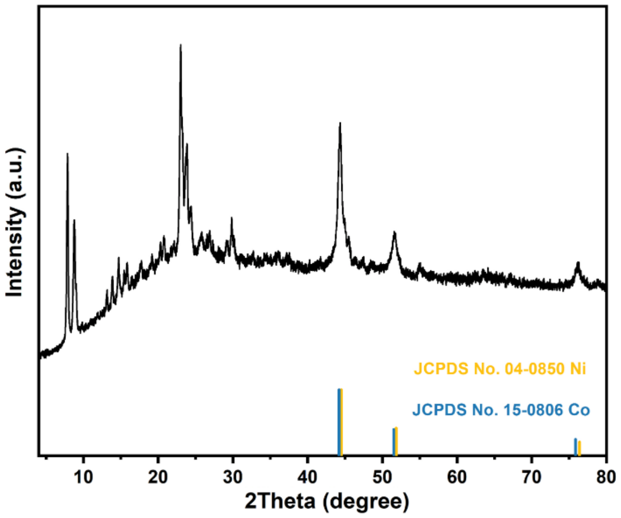

Figure S15. XRD pattern of ZSM-5@Ni-Co/SiO_2 NFs.

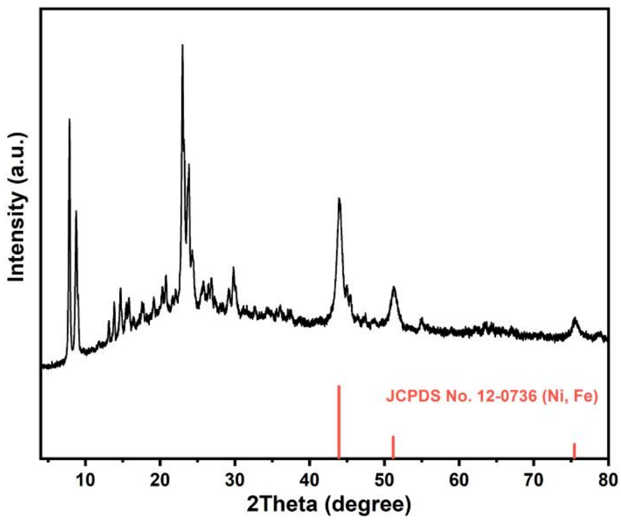

Figure S16. XRD pattern of ZSM-5@Ni-Fe/SiO_2 NFs.

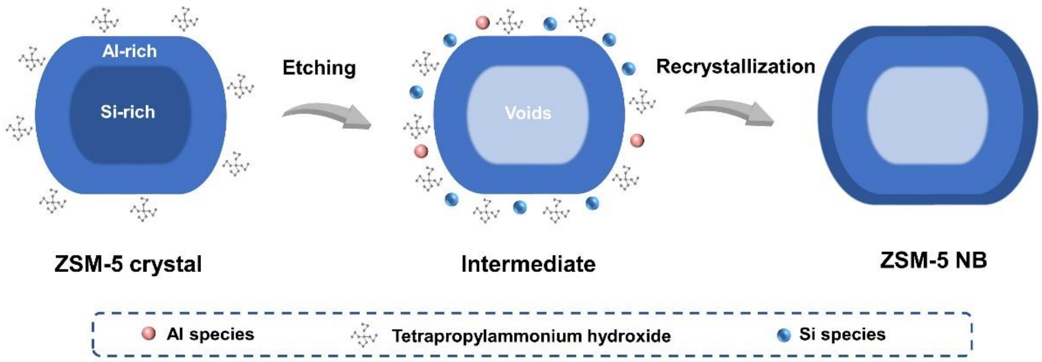

Figure S17. The detailed formation mechanism of ZSM-5 NB via etching-recrystallization transformation.

Note: ZSM-5 crystals are obtained using a conventional hydrothermal method. Typically, ZSM-5 crystals show inhomogeneous frameworks with Si-rich inner regions and Al-rich outer regions. Under alkaline conditions, the Si-rich part of the crystal is preferentially dissolved. Meanwhile, the dissolved species coassemble with OSDA to recrystallize on the surface of the Al-rich outer region, resulting in the formation of a hollow structure.

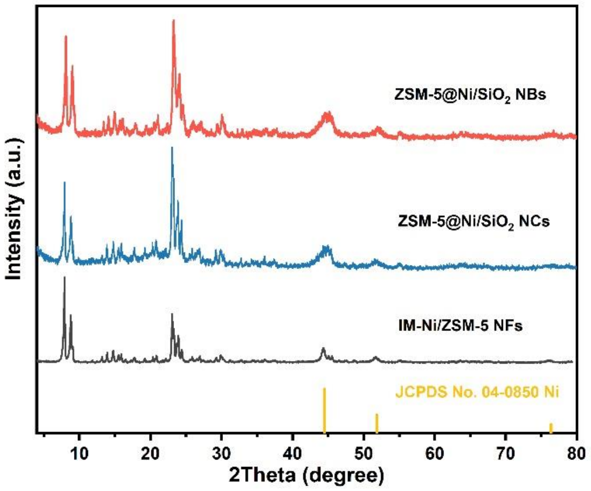

Figure S18. XRD patterns of ZSM-5@Ni/SiO_2 NBs, ZSM-5@Ni/SiO_2 NCs, and IM-Ni/ZSM-5 NFs.

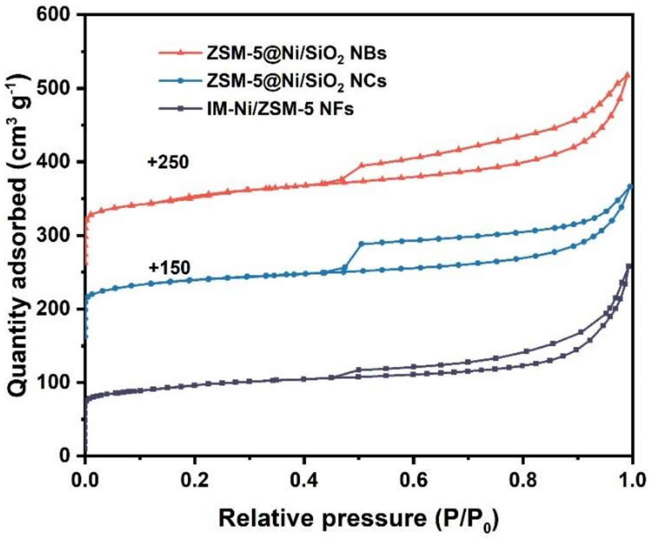

Figure S19. N_2 adsorption/desorption isotherms of ZSM-5@Ni/SiO_2 NBs, ZSM-5@Ni/SiO_2 NCs, and IM-Ni/ZSM-5 NFs.

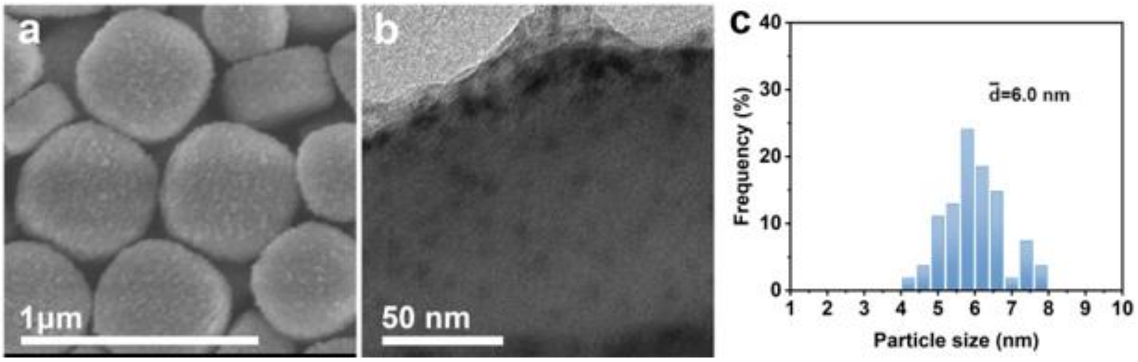

Figure S20. (a) SEM and (b) TEM images of ZSM-5@Ni/SiO_2 NCs and (c) the corresponding size distribution of Ni nanoparticles.

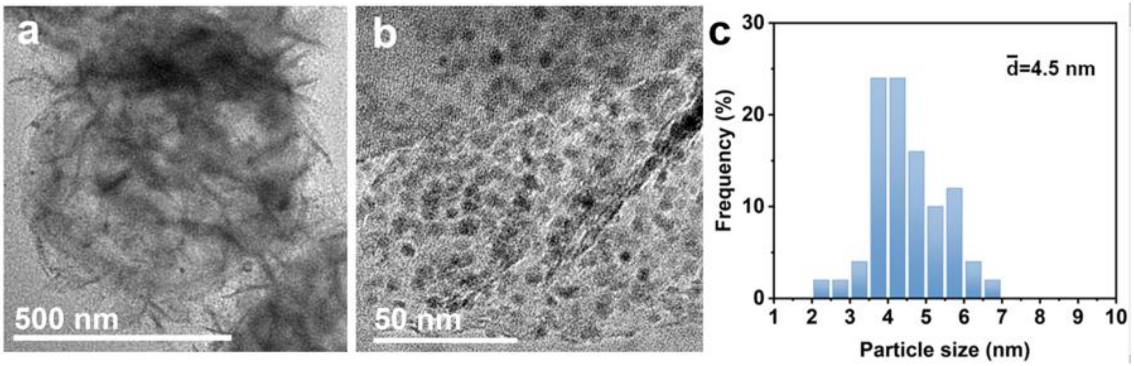

Figure S21. (a,b) TEM images of ZSM-5@Ni/SiO_2 NBs and (c) the corresponding size distribution of Ni nanoparticles.

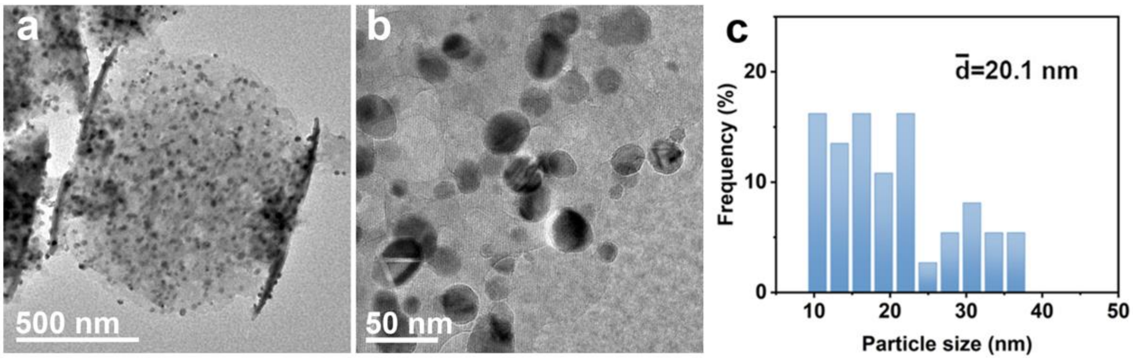

Figure S22. (a,b) TEM images of IM-Ni/ZSM-5 NFs and (c) the corresponding size distribution of Ni nanoparticles.

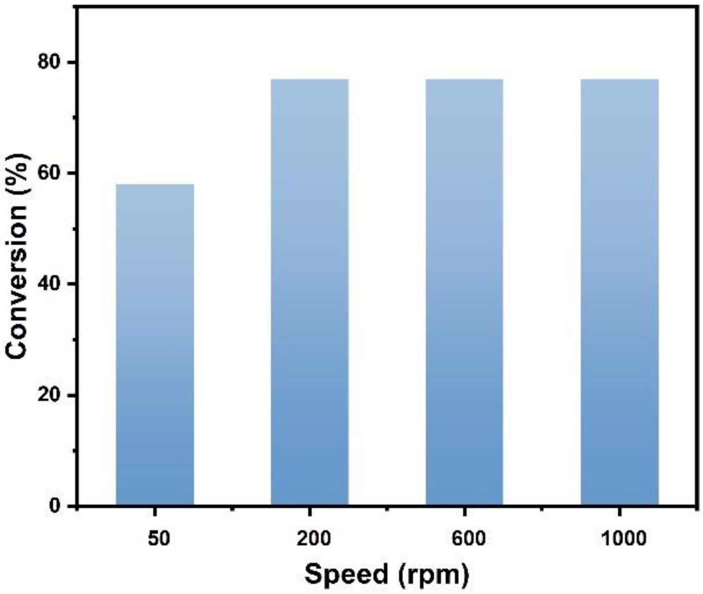

Figure S23. The effects of stirring speed on the conversion of stearic acid.

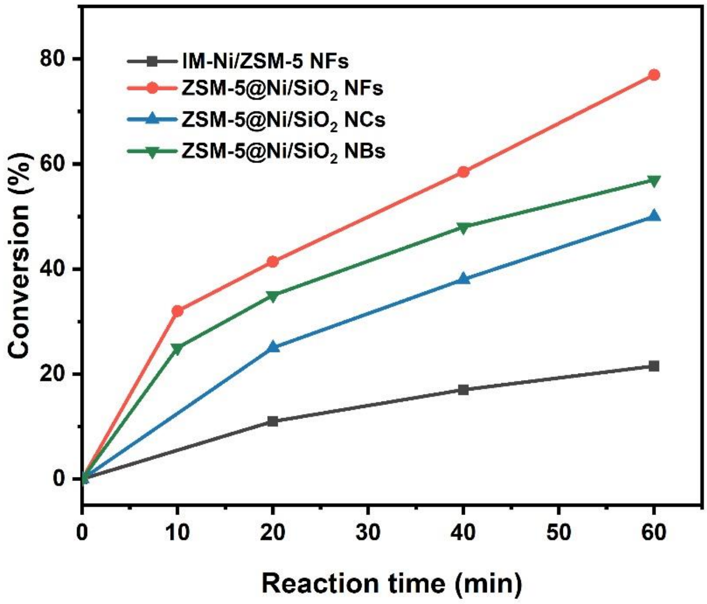

Figure S24. Conversion of stearic acid as a function of time over various prepared catalysts.

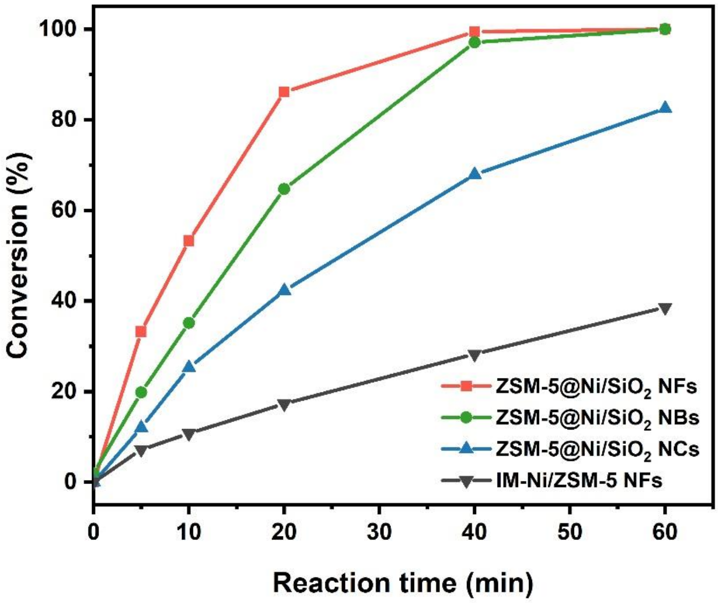

Figure S25. Conversion of 1-octadecanol as a function of time over various catalysts. Reaction conditions: 25 mg of catalysts, 1.0 g of 1-octadecanol, 40 ml of dodecane, reaction temperature of 260 °C, 40 bar of H_2, and stirring at 1000 rpm.

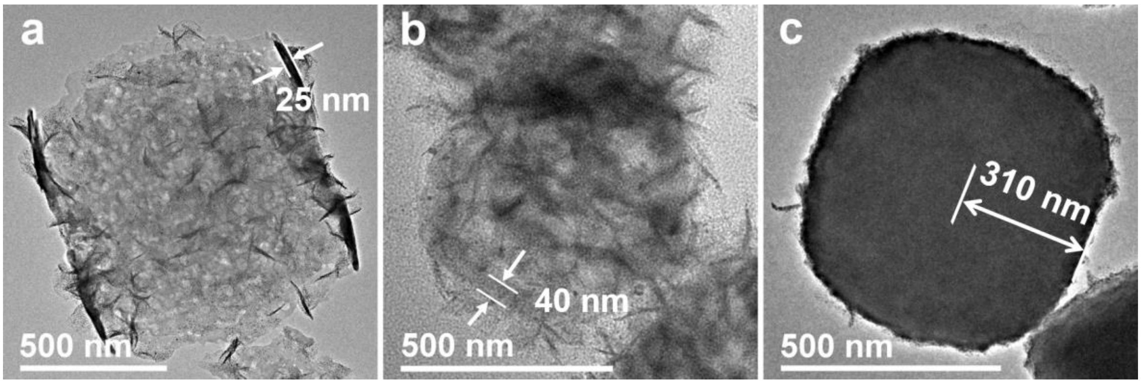

Figure S26. TEM images of the (a) ZSM-5@Ni/SiO_2 NFs, (b) ZSM-5@Ni/SiO_2 NBs, and (c) ZSM-5@Ni/SiO_2 NCs.

Table S1. The molar composition of the initial mixtures, temperature, and reaction time for the synthesis of various ZSM-5@metal silicate NFs.

Sample	Metal source	Metal /mmol	NH_3 /mmol	NH_4Cl /mmol	N_2H_4 /mmol	$Na_3C_6H_5O_7$ /mmol	Temperature / °C	Time /h
ZSM-5@Co silicate NFs	$Co(NO_3)_2·6H_2O$	0.25	3.5	2	0.27	0.10	90	6
ZSM-5@Fe silicate NFs	$FeSO_4·7H_2O$	0.14	4	2	0.27	0.25	80	4
ZSM-5@Ni-Co silicate NFs	$Co(NO_3)_2·6H_2O$	0.12	4	2	0.27	0.10	90	3
ZSM-5@Ni-Fe silicate NFs	$FeSO_4·7H_2O$	0.07	4	2	0.27	0.25	80	2

Table S2. Metal loading, average diameter, and dispersion of various catalysts.

Sample name	Ni loading [a] [wt%]	d_{Ni} [b] [nm]	D_{Ni} [c] [%]
ZSM-5@Ni/SiO_2 NFs	14.5	4.5	16
IM-Ni/ZSM-5 NFs	13.9	20.1	1

[a]The loading of Ni particles analyzed by inductively coupled plasma atomic emission spectroscopy (ICP-AES).

[b]The average diameter of Ni particles (d_{Ni}) measured based on TEM.

[c]The dispersion of Ni particles (D_{Ni}) calculated based on the CO pulse chemisorption.

Table S3. Acid site concentrations of ZSM-5@Ni/SiO_2 NFs and ZSM-5@Ni/SiO_2 NCs.

Sample name	BAS[a] [µmol/g]	External BAS[b] [µmol/g]	$f_{B,ext}$[c] (%)
ZSM-5@Ni/SiO_2 NFs	77	27	35
ZSM-5@Ni/SiO_2 NCs	58	7	12

[a]The number of Brønsted acid sites (BAS) determined by pyridine (Py) titration.

[b]The number of external Brønsted acid sites determined by 2,6-di-*tert*-butylpyridine (DTBP) titration.

[c]The fraction of external Brønsted acid sites calculated by (number of Brønsted acid sites by DTBP titration/number of Brønsted acid sites by Py titration)

Table S4. Comparison of normalized rates for the conversion of stearic acid and 1-octadecanol over various catalysts.

Sample name	Stearic acid conversion rate ($mol\ g^{-1}\ h^{-1}$)	1-octadecanol conversion rate ($mol\ g^{-1}\ h^{-1}$)
ZSM-5@Ni/SiO_2 NFs	0.466	4.069
ZSM-5@Ni/SiO_2 NBs	0.381	2.428
ZSM-5@Ni/SiO_2 NCs	0.175	1.469
IM-Ni/ZSM-5 NFs	0.084	0.881
